# Magnetic bead-based enhancement of qPCR detection rates in low-density *Plasmodium falciparum* samples: A comparative analysis of nucleic acid extraction methods

**DOI:** 10.1371/journal.pone.0355288

**Published:** 2026-08-06

**Authors:** Ruo-qiao Wen, Wei Ning, Fang Peng, Miao-miao Wang, Jie-peng Huang, Bin Tian

**Affiliations:** 1 The First Clinical Medical College, Inner Mongolia Medical University, Hohhot, Inner Mongolia, China; 2 Clinical Laboratory Department, Changsha Municipal Center for Disease Control and Prevention, Changsha, Hunan, China; 3 Xiangya School of Basic Medical Sciences, Central South University, Changsha, Hunan, China; Shanghai Jiao Tong University School of Medicine, CHINA

## Abstract

**Background:**

In post-elimination malaria settings, monitoring asymptomatic low-density infections following Artemisinin-based Combination Therapy (ACT) is critical for interrupting occult transmission and alerting to drug resistance. Real-time quantitative PCR (qPCR) is central to molecular surveillance, yet its sensitivity depends on extracted DNA quality. This study evaluated three DNA extraction methods to provide a basis for optimizing laboratory screening protocols for low-density *Plasmodium falciparum (P. falciparum)* infections.

**Methods:**

Nucleic acids were extracted from *P. falciparum-positive* whole blood samples using a silica membrane spin column method (M1), a modified red blood cell lysis method (M2), and a magnetic bead-based method (M3). DNA concentration and purity (A260/A280 ratio) were measured, and integrity assessed via agarose gel electrophoresis. Template yield and amplification efficiency were compared by qPCR. Furthermore, the detection sensitivity was compared between M1 and M3 via serial dilution experiments. Clinical performance was validated using 40 microscopy-negative or low-density (<150 parasites/µL) samples collected 48 hours post-treatment.

**Results:**

M3 yielded significantly higher DNA concentration (39.0 ± 7.57 ng/µL) than M1 (10.49 ± 2.73 ng/µL) and M2 (7.48 ± 1.76 ng/µL) (*P* < 0.0001). Although M3 had a slightly lower A260/A280 ratio, electrophoresis showed it better preserved high-molecular-weight DNA. qPCR confirmed that the template yield from M3 was approximately 4.0-fold and 2.8-fold higher than that from M2 and M1, respectively. Serial dilution experiments demonstrated that M3 exhibited significantly higher detection sensitivity, with a lower limit of detection (LOD) than M1. Among the 40 clinical low-density samples, the positive detection rate for M3 (77.5%, 31/40) was significantly higher than for M1 (60.0%, 24/40) (*P* = 0.0082), with M1 showing a missed detection rate of 22.6% (7/31).

**Conclusion:**

The magnetic bead-based method (M3) exhibits marked advantages in DNA yield, DNA integrity and downstream qPCR sensitivity, which elevates the detection rate of low-density *P. falciparum* and reduces missed detection risk. For post-elimination surveillance and screening of asymptomatic infections, prioritizing magnetic bead-based nucleic acid extraction can provide more reliable technical support for molecular surveillance in post‑elimination settings.

## Introduction

Malaria remains a major global public health challenge. The World Malaria Report 2025 estimates 282 million cases and nearly 610,000 deaths annually, predominantly due to *Plasmodium falciparum* (*P. falciparum*) [1]. Despite China’s malaria-free certification in 2021 [2], persistent risks from imported cases are underscored by a 274% increase in reports from 2022 to 2024 [3,4]. Globally, local transmission of malaria re-emerged in multiple U.S. states in 2023 after an absence of over two decades [5,6]. Countries including Spain [7], Greece [8], and Sri Lanka [9] have also reported non-imported malaria cases within the past decade. This evolving landscape underscores the urgent need for more sensitive malaria surveillance systems.

In the current post-elimination phase, malaria surveillance faces dual challenges. The first is the accurate identification of asymptomatic, low-density infections to interrupt cryptic transmission [10]. In areas with reduced transmission intensity, the infection profile shifts towards low-density and asymptomatic presentations [11–13]. In such individuals, peripheral blood parasite densities are often below 50 parasites/μL [11], resulting in extremely low positivity rates by microscopy and posing a significant challenge for laboratory detection. The second challenge is the timely detection and monitoring of artemisinin resistance. Artemisinin-based combination therapy (ACT) is the cornerstone of global malaria treatment and control. However, the emergence of artemisinin resistance in Southeast Asia poses a serious threat to its long-term efficacy. Multiple studies have reported the detection of residual submicroscopic parasitemia by highly sensitive polymerase chain reaction (PCR) on day 3 following ACT treatment [14–16]. These residual parasites may indicate tolerance to artemisinin and represent a potential source for treatment failure, recrudescence, and the spread of resistance [17]. Therefore, sensitive monitoring of post-ACT residual low-density infections has become a critical component for evaluating therapeutic efficacy and providing early warning of drug resistance.

Malaria diagnosis in primary healthcare institutions currently relies primarily on Rapid Diagnostic Tests (RDTs) and microscopic examination. While RDTs offer rapid operation and convenience, their accuracy can be compromised by limitations such as persistent antigenemia or deletions of the target pfhrp2/3 genes [18]. Microscopy, despite its high specificity and low cost, is highly dependent on well-trained technicians. A prior systematic evaluation of local microscopy competency by our research team revealed that microscopy still carries risks of missed detection and misidentification for low-density infections and non-typical *Plasmodium* species [19]. To address this challenge, our team explored and validated the short-term effectiveness of a standardized training pathway to enhance microscopists’ competency [20]. However, we recognize that training cannot fundamentally alter the inherent limitations of microscopy: its finite sensitivity (10–100 parasites/μL) [21] and high dependence on subjective expertise. These intrinsic drawbacks of conventional methods, which readily lead to missed diagnoses of low-density samples and failure to detect asymptomatic carriers, have become a critical technical bottleneck for consolidating elimination gains and interrupting cryptic transmission chains.

Molecular techniques such as real-time quantitative PCR (qPCR) provide the high sensitivity (5–10 parasites/μL) and specificity necessary for detecting low-density infections and monitoring drug resistance [10,22–24]. However, qPCR performance is critically dependent on template DNA quality [25]. Its concentration, purity, and integrity directly determine the success and efficiency of downstream amplification reactions. Commonly used DNA extraction methods, including silica membrane columns, modified red blood cell lysis, and magnetic bead-based protocols, vary in their principles, workflows, and effects on nucleic acid yield, purity, and integrity. A systematic comparison of their efficacy in extracting *P. falciparum* DNA and the subsequent impact on qPCR sensitivity is currently lacking but essential for optimizing molecular detection protocols in post-elimination settings.

Therefore, this study systematically evaluated these three methods, comparing their DNA yield, purity, and integrity from *P. falciparum*-positive blood, and assessing their impact on qPCR sensitivity. The aim is to provide an experimental basis for establishing a more standardized molecular detection protocol suitable for post-elimination surveillance. This work holds important practical significance for consolidating elimination achievements and addressing the threat of drug resistance.

## Materials and methods

### Sample collection and study design

Whole blood samples from *P. falciparum* infections required for this study were provided by the Changsha Municipal Center for Disease Control and Prevention. The samples were accessed for research purposes on 15 January 2026. All sample collection strictly adhered to clinical ethics protocols. The authors did not have access to information that could identify individual participants during or after data collection, as all samples were fully anonymized prior to receipt. Based on the experimental objectives, samples were categorized into three groups:

#### Samples for performance comparison.

Ten whole blood samples (S1-S10) from patients with acute *P. falciparum* malaria were selected to compare three nucleic acid extraction methods (M1: silica membrane spin column, M2: modified red blood cell lysis, M3: magnetic bead-based method) for differences in nucleic acid yield, purity, and integrity.

#### Clinical validation samples.

Forty EDTA-anticoagulated blood samples were collected from clinically confirmed *P. falciparum* patients 48 hours after receiving standard treatment. All samples were re-examined by expert microscopy of thick and thin blood smears and were confirmed to be either negative or have a parasite density <150 parasites/μL. These were used to evaluate the detection efficacy of the M1 and M3 methods on actual low-density samples.

#### Samples for sensitivity assessment.

Fresh whole blood from a patient with acute *P. falciparum* malaria was used. Based on parasite counts from thick and thin blood smears, the sample was serially diluted (10-fold from 10^1^ to 10^6^) using uninfected human whole blood to create a concentration gradient. The uninfected blood used for dilution was obtained from a healthy volunteer who had no recent travel history to malaria-endemic areas and no known prior episode of malaria. The blood was further confirmed to be negative for *P. falciparum* by both expert microscopy of thick and thin blood smears and qPCR. This gradient was used to determine the limit of detection (LOD) for the M1 and M3 methods, defined as the lowest parasite density (in parasites/μL) that yielded consistent positive amplification in triplicate tests. Each dilution was tested in triplicate.

### DNA extraction methods

Three different DNA extraction methods were applied in parallel to the samples: the silica membrane spin column method (M1), the modified red blood cell lysis method (M2), and the magnetic bead-based method (M3). All methods started with 50 μL of EDTA-anticoagulated whole blood and used a final elution volume of 50 μL.

#### Key reagents and equipment.

- M1 (Silica Membrane Spin Column): The Thermo Scientific GeneJET Genomic DNA Purification Kit (Cat. No. 01161293) was used according to the manufacturer’s instructions. Key equipment included a thermomixer and a microcentrifuge.
- M2 (Modified Red Blood Cell Lysis): Red blood cells were lysed using sterile deionized water. Subsequent DNA purification steps were identical to M1, using the same kit.
- M3 (Magnetic Beads): The TianLong Bio GeneRotex96 Nucleic Acid Extraction and Purification Kit (Magnetic Bead Method) was used, along with its dedicated magnetic stirring rods and an 80°C metal bath.

#### Overview of operational procedures.

The detailed operational steps and key parameters for the three methods are summarized and compared in Table 1.

**Table 1:** Detailed operational procedures of the three DNA extraction methods.

Experimental Step	M1: Silica Membrane Spin Column	M2: Modified RBC Lysis	M3: Magnetic Beads
**I. Sample Pre-treatment**	None	I. Add 1 mL deionized water for RBC lysis, incubate at room temperature for 5 min. II. Centrifuge at 13,000 × g, 4°C for 5 min, discard supernatant. III. Repeat steps I-II once.	None
**II. Lysis**	I. Add 400 μL Lysis Buffer and 20 μL Proteinase K. II. Incubate at 56°C for 10 min.	For post-lysis pellet: Same as M1 steps.	I. Add 50 μL Binding Solution, 50 μL Lysis Buffer, and 15 μL Proteinase K. II. Lyse in an 80°C metal bath for 5 min.
**III. Nucleic Acid Binding**	I. Add 200 μL absolute ethanol, mix. II. Transfer all liquid to purification column. III. Centrifuge at 6,000 × g for 1 min (binding).	Same as M1 steps.	I. Insert magnetic stirring rod. II. Stir continuously at 80°C for 15 min (adsorption).
**IV. Washing**	I. Add 500 μL Wash Buffer I, centrifuge at 8,000 × g for 1 min. II. Add 700 μL Wash Buffer II, centrifuge at 12,000 × g for 3 min.	Same as M1 steps.	Magnetic rod transfer to wash buffer wells: I. Wash in Wash Buffer I for 3 min. II. Wash in Wash Buffer II at 80°C for 2 min. III. Quick wash in Wash Buffer III for 10 sec.
**V. Elution**	I. Transfer column to a new tube. II. Add 50 μL pre-warmed Elution Buffer to the center of the membrane. III. Stand at room temperature for 2 min. IV. Centrifuge at 8,000 × g for 1 min.	Same as M1 steps.	I. Transfer magnetic rod to 50 μL Elution Buffer. II. Incubate at room temperature for 5 min. III. Remove the rod to obtain the eluate.
**VI. Storage**	−20°C	−20°C	−20°C

### Preparation, staining, and parasite counting of thick and thin blood smears

Fresh *P. falciparum* whole blood was used to prepare thick and thin blood smears. Thin smears were prepared using the push-slide method, air-dried, and fixed with methanol for 30 seconds. Thick smears were air-dried (not fixed), avoiding hemolysis. Both smears were stained with 10% Giemsa stain at room temperature for 30 minutes, rinsed gently with saline, and air-dried. Smears were examined under oil immersion microscopy. Parasites were counted per 200 white blood cell (WBC) fields, and the peripheral blood parasite density (parasites/μL) was calculated assuming 8,000 WBCs/μL of blood. These counts served as the basis for the serial dilutions in the sensitivity experiments. All parasite counts were performed by a single, WHO-certified Level 1 malaria microscopist. All readings were conducted in a blinded manner, without knowledge of sample identity or experimental group. To minimize intra-observer variability, each slide was counted three times, and the final parasite density was calculated as the mean of the three counts. The microscopist regularly participates in external quality assurance programs for malaria microscopy.

### Agarose gel electrophoresis analysis

A 1.5% agarose gel was prepared by mixing agarose with ethidium bromide (EB), pouring it into a casting tray, and allowing it to solidify. For each DNA extraction method, 5 μL of the extracted DNA was mixed with 1 μL of Loading Buffer and loaded into the gel wells alongside a DL2000 DNA Marker as a molecular weight reference. Electrophoresis was performed at a constant voltage of 120 V for 30 minutes. After electrophoresis, gels were visualized and photographed using a gel imaging system to analyze DNA band distribution, integrity, and degradation. The FastPure Gel DNA Extraction Mini Kit (Vazyme, Cat. No. DC301-01) was used to recover large and small nucleic acid fragments from the gel. The recovered products were stored at −20°C for subsequent PCR validation.

### Real-time quantitative PCR (qPCR) detection

The *P. falciparum* Nucleic Acid Detection Kit (Sansure Biotech, Cat. No. YJW20103N) was used with the Roche LightCycler 480 II qPCR system. This qPCR assay targets the multi-copy 18S rRNA gene of *P. falciparum* and employs SYBR Green-based fluorescent chemistry. The total reaction volume was 25 μL, prepared strictly according to the kit instructions: containing 20 μL of the kit-provided master mix and 5 μL of the test DNA sample (or recovered nucleic acid samples, negative/positive controls). The amplification conditions were: 95°C for 5 min (pre-denaturation); followed by 40 cycles of 95°C for 10 sec (denaturation) and 58°C for 30 sec (annealing/extension with fluorescence acquisition). The oligonucleotide primers used were: PFL, 5′-TTATTATCCTTTGATTTTTATCTTTGG-3′; PFR, 5′-ATAAATTTATTACGTGTTACTTCTTTG-3′.

### Nucleic acid purity assessment

The purity of DNA samples extracted by the three methods was assessed using a NanoDrop™ One spectrophotometer (Thermo Fisher Scientific) by measuring the absorbance ratio at 260 nm and 280 nm (A_260_/A_280_). An A_260_/A_280_ ratio between 1.7 and 1.9 was considered indicative of high-quality DNA.

### Experiment on the effect of lysis time on nucleic acid yield

To analyze the effect of lysis time on DNA yield, four lysis time points (10, 30, 60, and 120 minutes) were tested. The M1 method was used to extract nucleic acids from the same batch of *P. falciparum* whole blood samples, with each time point tested in triplicate. Following extraction, qPCR was performed, and the resulting Ct values were analyzed to assess the impact of lysis duration.

### Statistical analysis

Statistical analysis and graph plotting were performed using GraphPad Prism 9 software. Measurement data are presented as mean ± standard deviation (x ± s), and enumeration data as number (n) and percentage (%). The Shapiro-Wilk test was first used to assess data normality. For normally distributed quantitative data (e.g., DNA concentration, A_260_/A_280_ ratio, Ct values): comparisons among three or more paired groups (e.g., performance of M1, M2, M3) were performed using repeated-measures analysis of variance (ANOVA), with post-hoc multiple comparisons conducted using the Bonferroni method; comparisons between two paired groups (e.g., Ct values of M1 vs. M3 in dilution experiments) were performed using the paired samples t-test. For enumeration data (e.g., positive detection rates), the consistency of results between two methods was compared using McNemar’s test for paired 2x2 tables. A *P*-value < 0.05 was considered statistically significant for all analyses.

## Results

### Comparison of DNA yield, purity, and amplification efficiency

**Fig 1:**
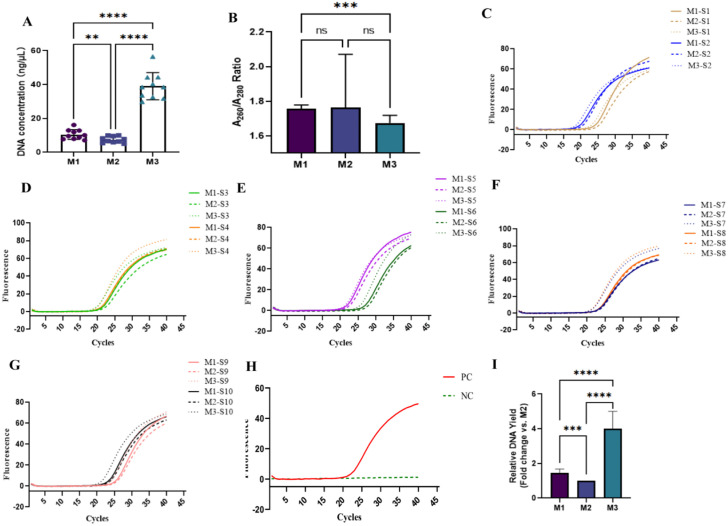
DNA yield, purity, and qPCR amplification efficiency. (A) DNA concentrations by M1, M2, and M3 (n = 10). (B) A_260_/A_280_ ratios. (C–G) qPCR amplification kinetics curves. (H) Amplification kinetics curves for positive and negative controls. (I) Relative template recovery efficiency (2^–ΔCt^) with M2 set as the baseline (value of 1). For all quantitative panels (A, B, I), data are presented as mean ± standard deviation (mean ± SD). Statistical significance was assessed by repeated-measures ANOVA with Bonferroni correction (ns indicates no significant statistically, **P < 0.01, ***P < 0.001, ****P < 0.0001).

DNA concentration differed significantly among the three methods (Fig 1A). The magnetic bead-based method (M3) yielded the highest DNA concentration (39.0 ± 7.57 ng/μL), significantly surpassing both the silica membrane spin column method (M1, 10.49 ± 2.73 ng/μL, *P* < 0.0001) and the modified RBC lysis method (M2, 7.48 ± 1.76 ng/μL, *P <* 0.0001). M1 also outperformed M2 (*P <* 0.01). The low yield of M2 is attributed to the loss of nucleic acids during repeated distilled water lysis and centrifugation steps, while M3’s superior performance stems from the specific adsorption of nucleic acid onto magnetic beads within the complex whole blood matrix, minimizing loss by avoiding repeated supernatant removal.

In DNA purity assessment (Fig 1B), the A_260_/A_280_ ratios for M1 (1.75 ± 0.02) and M2 (1.76 ± 0.31) were within the optimal 1.7–1.9 range, indicating effective removal of protein contaminants. The ratio for M3 (1.67 ± 0.04) was slightly lower, which may be attributed to minor co-adsorption of host proteins during the magnetic bead incubation that were not fully removed in subsequent washes.

The practical applicability of the extracted DNA was evaluated via qPCR amplification of the *P. falciparum* target gene. Amplification kinetics curves (Fig 1C–G) confirmed successful amplification for all methods, showing typical sigmoidal shapes. However, amplification efficiency varied notably. Reactions using M3-derived templates (dotted lines) exhibited the sharpest inflection points, the fastest fluorescence increase rates, and the highest plateau fluorescence values in representative samples (S3, S4, S7, S8, S9, S10), indicating optimal amplification efficiency despite the slight protein carryover. Templates from M1 (solid lines) showed slightly inferior performance in these parameters, while those from M2 (dashed lines) generally demonstrated the slowest fluorescence rise and the lowest plateau values in samples S1–S6. Positive and negative controls (Fig 1H) showed expected amplification and no signal, respectively, confirming assay specificity and reliability.

Quantitative analysis of relative template recovery efficiency (2^–ΔCt^) using M2 as baseline (1.00) further corroborated these findings (Fig 1I). M1 yielded a 1.45 ± 0.22-fold increase over M2, while M3 achieved a 4.03 ± 1.06-fold increase, equivalent to a 2.8-fold advantage over M1. These results collectively indicate that M3 provides the highest yield of high-quality, amplifiable *P. falciparum* DNA, and this magnitude of yield difference can directly influence whether a low-density infection is detected.

### Effect of different lysis durations on DNA extraction yield

**Fig 2:**
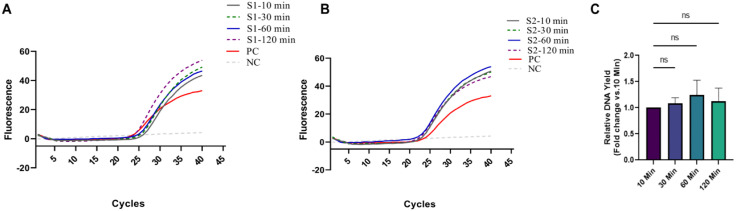
Effect of lysis durations on DNA extraction yield and amplification efficiency. (A, B) qPCR amplification curves for two representative samples after lysis for 10, 30, 60, or 120 min (M1). (C) Relative template yield (2^-ΔCt^) with the 10-minute group as baseline, from three independent experiments. Data are mean ± SD. NS indicates no statistical significance (*P* > 0.05).

To investigate the operational parameters potentially contributing to the significantly lower nucleic acid yields of M1 and M2 compared to M3, we systematically evaluated a key shared variable: lysis time. Whole blood samples from *P. falciparum* patients were subjected to four lysis durations (10, 30, 60, and 120 minutes) prior to DNA extraction and qPCR analysis. As shown in the representative qPCR amplification kinetics curves for Sample 1 (Fig 2A) and Sample 2 (Fig 2B), all lysis time groups produced amplification curves with typical sigmoidal shapes. No discernible differences were observed in curve inflection sharpness or fluorescence rise rate across the different lysis times, indicating neither an improvement nor a decline in amplification performance with prolonged lysis. Quantitative analysis of relative template yield (2^−ΔCt^), using the 10-minute lysis group as the baseline, further confirmed these observations (Fig 2C). For all samples tested, no statistically significant differences in DNA yield were detected among the different lysis duration groups (*P >* 0.05). These results demonstrate that varying the lysis time within a broad range of 10–120 minutes did not significantly affect DNA recovery efficiency or subsequent qPCR amplification performance under the conditions of this study.

### Detection performance of M1 and M3 methods in simulated low-parasite-load samples

To compare the sensitivity of M1 and M3 for low parasite densities, a ten-fold serial dilution series (from 10^1^ to 10^6^) was prepared from four *P. falciparum*-positive whole blood samples with known parasite counts (determined by microscopy). This created a concentration gradient spanning several orders of magnitude, simulating clinical samples with varying parasite loads. Parallel DNA extraction and qPCR detection were then performed using both methods.

**Fig 3:**
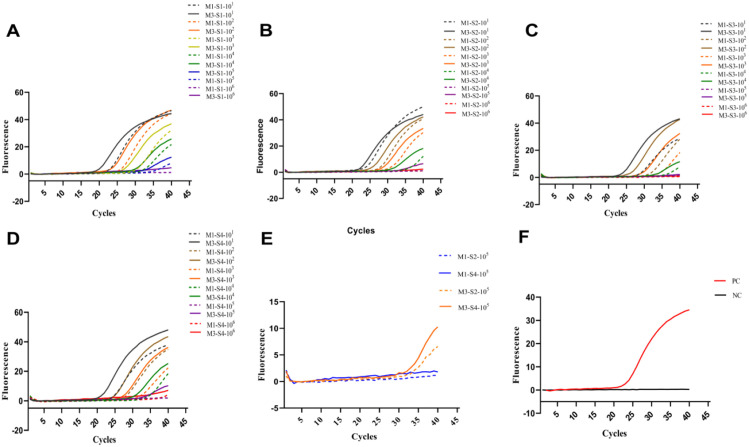
Comparison of qPCR detection performance between M1 and M3 methods for serially diluted *P. falciparum* samples. (A–D) qPCR amplification curves of DNA extracted from Samples 1 to 4 after serial dilution (10^1^- to 10^6^-fold) using M1(dashed) and M3(solid). Each dilution was tested in triplicate. (E) Direct comparison of amplification curves for Samples 2 and 4 at the 10^5^-fold dilution. (F) Amplification curves for the negative and positive control.

The qPCR results (Fig 3A-F) demonstrated a significant advantage for the M3 method in detecting low-parasite-load samples, evident both in quantitative Ct values and visually in the amplification kinetics. As shown in Fig 3A-D, for high-dilution samples, the amplification curves for the M3 method (solid lines) exhibited earlier inflection points, steeper rising slopes, and higher plateau fluorescence intensities—characteristic features indicative of higher initial template quantity and superior amplification efficiency. In contrast, the M1 method (dashed lines) showed markedly delayed signal increase and lower plateaus at equivalent dilutions. These trends are quantified in Table 2. Across the four samples, M3 detected *P. falciparum* DNA in three of four samples at the 10^5^ dilution, whereas M1 detected none at this level. At lower dilutions (10^1^–10^3^), both methods yielded consistently positive detections across all four samples, as expected given the high parasite densities at these levels. The discriminatory power between the two methods emerged at the 10^4^–10^6^ dilutions. Specifically, for Samples 2 and 4, the M3 method still produced typical sigmoidal amplification curves at a 10^5^ dilution (theoretical densities: 17.9–27.4 parasites/μL; Fig 3E), whereas the M1 method yielded only faint or no detectable amplification signals at this dilution, indistinguishable from background noise.

**Table 2:** qPCR detection results of M1 and M3 methods at all dilution levels (10^1^–10^6^).

Sample	Original Density	Dilution (Theoretical Density)	M1	M3
1	7.97 × 10^6^	10^1^ (797000)	+	+
		10^2^ (79700)	+	+
		10^3^ (7970)	+	+
		10^4^ (797)	+	+
		10^5^ (79.7)	–	+
		10^6^ (8.0)	–	–
2	2.74 × 10^6^	10^1^ (274000)	+	+
		10^2^ (27400)	+	+
		10^3^ (2740)	+	+
		10^4^ (274)	+	+
		10^5^ (27.4)	–	+
		10^6^ (2.74)	–	–
3	2.44 × 10^5^	10^1^ (24400)	+	+
		10^2^ (2440)	+	+
		10^3^ (244)	+	+
		10^4^ (24.4)	+	+
		10^5^ (2.4)	–	–
		10^6^ (0.24)	–	–
4	1.79 × 10^6^	10^1^ (179000)	+	+
		10^2^ (17900)	+	+
		10^3^ (1790)	+	+
		10^4^ (179)	+	+
		10^5^ (17.9)	–	+
		10^6^ (1.79)	–	–

+, positive detection; –, negative detection.

A key and noteworthy finding was observed with Sample 3, which had the lowest original parasite density (2.44 × 10^5^ parasites/μL). Both M1 and M3 methods generated clear amplification curves at the 10^4^ dilution (24.4 parasites/μL; Fig 3C, Table 2), indicating that both methods recovered sufficient template for effective PCR amplification in this specific sample. This contrasts sharply with the earlier failure of the M1 method in Samples 1, 2, and 4, suggesting that sample-specific matrix or characteristics might significantly influence the extraction efficiency of the column-based method (M1), whereas the magnetic bead-based method (M3) demonstrated greater robustness and consistency.

### DNA integrity and functional analysis via gel electrophoresis and fragment recovery

To compare the integrity and function of nucleic acids extracted by the three methods, products from six samples (S1–S6) were analyzed by agarose gel electrophoresis, with specific bands recovered and validated by qPCR.

**Fig 4:**
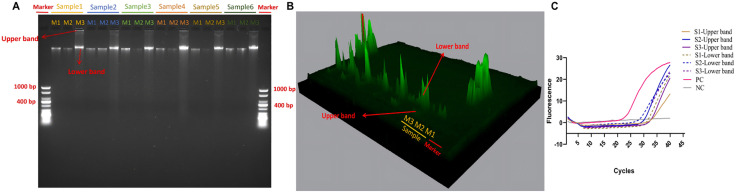
DNA integrity and functional validation. (A) Agarose gel. Lane 1: DNA ladder. For each sample (e.g., lanes 2–4 for S1), products from M1, M2, and M3 are shown. M3 displays Upper (UB) and Lower (LB) bands. (B) Fluorescence intensity profile. (C) qPCR amplification curves of gel-purified UB and LB from M3 extracts (S1–S3), confirming template activity.

Electrophoresis results (Fig 4A) revealed a distinct fragment profile for M3. All six samples extracted by M3 showed a unique, diffuse high-molecular-weight band (Upper Band, UB) near the wells, along with a clear band (Lower Band, LB) comigrating with the primary band seen in M1 and M2 extracts. In contrast, M1 and M2 extracts typically displayed only a single band corresponding to the LB, with generally weaker intensity than the LB from M3. Fluorescence intensity analysis (Fig 4B) quantified this difference, showing stronger signals in both the UB and LB regions for M3, confirming higher total DNA recovery and better preservation of genomic integrity.

To test the template activity of these fragments, the UB and LB from M3 extracts of samples S1–S3 were gel-purified and subjected to the same *P. falciparum*-specific qPCR. Both recovered fractions produced typical sigmoidal amplification curves (Fig 4C), confirming that they contained intact target sequences and were functional templates. Although the UB showed slightly higher Ct values—likely due to lower recovery efficiency of large fragments—this result proves that the high-molecular-weight DNA preserved by M3 is amplifiable.

In summary, M3 not only yielded more total DNA but also uniquely preserved both high-molecular-weight genomic DNA (UB) and the more readily amplified main fragment (LB). M1 and M2 primarily recovered the latter, with lower efficiency. This difference in both DNA quantity and integrity underpins the superior qPCR sensitivity of the M3 method.

### Validation of M1 and M3 performance in low-density clinical samples

To evaluate M1 (column-based) and M3 (magnetic bead-based) methods in a real-world, low-parasitemia context, we collected 40 whole-blood samples from confirmed *P. falciparum* patients 48 hours after standard treatment. All post-treatment samples were confirmed by microscopy to be either negative or contain <150 parasites/μL, forming a low-density validation set. DNA was extracted in parallel using M1 and M3 and detected by the same qPCR assay.

As summarized in Table 3, M3 achieved a significantly higher positive detection rate (77.5%, 31/40) than M1 (60.0%, 24/40) (McNemar’s test, χ^2^ = 7.00, *P* = 0.0082). Using M3 as the reference, M1 failed to detect 22.6% (7/31) of positive cases. This 22.6% missed detection rate highlights that reliance on M1 alone would miss over one-fifth of low-density infections in surveillance.

**Table 3:** Parallel detection results of M1 and M3 methods for 40 low-density clinical samples.

	M1 Positive	M1 Negative	Total
**M3 Positive**	24	7	31
**M3 Negative**	0	9	9
**Total**	24	16	40

These clinical results align with our controlled dilution experiments (*Detection Performance of M1 and M3 Methods in Simulated Low-Parasite-Load Samples*), confirming that the magnetic bead-based method (M3) provides superior detection of low-density *P. falciparum* infections due to its higher DNA yield and recovery. M3 thus offers a more reliable solution for accurate surveillance in post-elimination settings.

## Discussion

This study systematically evaluated the performance of three DNA extraction methods—silica membrane spin column (M1), modified red blood cell lysis (M2), and magnetic bead-based (M3)—from *P. falciparum* whole blood, with a focus on their utility for low-density infection detection. The results demonstrate that the magnetic bead-based method (M3) exhibits consistent and significant advantages in nucleic acid yield, DNA integrity, and downstream qPCR sensitivity. Specifically, M3 yielded the highest DNA concentration, effectively preserved high-molecular-weight fragments, and achieved significantly higher positive detection rates than the conventional column-based method (M1) in both simulated dilution series and real-world low-density clinical samples. These findings collectively indicate that optimizing the upstream nucleic acid extraction step is crucial for enhancing the sensitivity of molecular diagnosis for low-density *P. falciparum* infections. This study provides direct experimental support for establishing higher-sensitivity screening protocols for asymptomatic and low-density infections in post-elimination surveillance systems.

Regarding the superior recovery of parasite DNA by the magnetic bead method, our findings are consistent with Holzschuh et al. [26], who reported a 3–5-fold higher recovery efficiency of *P. falciparum* DNA from whole blood using magnetic beads compared to silica columns. We observed a 2.8-fold average qPCR template advantage for M3 over M1. In the study by Holzschuh et al., recovery efficiency was quantified by ddPCR targeting a single-copy gene (tRNA), which directly reflects the proportion of parasite genomes recovered. Our study used a commercial qPCR assay targeting the multi-copy 18S rRNA gene for both template quantification and detection. Because the 18S rRNA gene is multi-copy, the relative template estimates reported here cannot be directly compared to single-copy genome recovery rates. Nevertheless, both our dilution experiments (where M3 reliably detected parasites at approximately 20 parasites/μL while M1 failed) and clinical validation (77.5% detection by M3 vs. 60.0% by M1) confirm that the performance advantage of magnetic bead extraction translates into clinically meaningful gains in sensitivity and reduced missed detection, anchoring the methodological comparison in a pragmatically relevant context.

Furthermore, our results complement and are consistent with studies on other pathogens: Na et al. [27] and Farani et al. [28] reported advantages of magnetic bead methods for extracting bacterial and Trypanosoma cruzi DNA in sepsis and Chagas disease research, respectively. Notably, Lorente-Leal et al. [29] observed that magnetic bead extraction might yield slightly lower DNA amounts than column methods in bovine tuberculosis tissue samples, suggesting that extraction efficiency can be influenced by sample type (whole blood vs. tissue) and specific lysis protocols. This underscores the need for context-specific validation.

This study focused on the critical step of genomic DNA recovery from low-density blood samples, particularly from post-ACT clinical samples with extremely low template levels that may include atypical parasites under drug pressure. The clinical validation confirms that the magnetic bead method maintains high detection rates even in such challenging samples. Its 17.5% absolute increase in detection rate over the column method enables more sensitive capture of residual infection signals that may indicate suboptimal treatment response or potential drug resistance. This magnitude of improvement means that approximately one additional low-density infection is identified for every six patients tested, reducing the likelihood that residual parasitaemia goes undetected.

The advantages of M3 in yield and integrity stem from its specific purification principle and standardized workflow. Unlike column methods (M1/M2), which rely on nonspecific silica adsorption and involve multiple centrifugation and transfer steps, M3 is based on specific binding of nucleic acids to functional groups (e.g., silanol or carboxyl) coated on magnetic beads. This process is performed in closed tubes or on automated platforms, where gentle lysis releases nucleic acids, and magnetic separation minimizes physical shearing and template loss associated with repeated pipetting, centrifugation, and column transfers. Our results visually support this principle: agarose gel electrophoresis showed that M3 better preserved high-molecular-weight DNA, and qPCR quantification confirmed that its template yield was over four times higher than that of the traditional lysis-based method. By enabling efficient and gentle capture, M3 fundamentally improves the recovery of low-abundance parasite DNA.

A noteworthy observation is that DNA extracted by M3 had a slightly lower A_260_/A_280_ ratio (1.67 ± 0.04) than that from M1/M2, falling just below the classic “high-quality” range of 1.7–1.9. The A260/A280 ratio, while a convenient preliminary indicator, can be influenced by residual buffer components, salts, or specific lysis products [30]. A lower ratio typically suggests protein contamination. For M3, the slightly reduced ratio may result from its gentle lysis and wash protocols, which maximize nucleic acid recovery and preservation but may allow minimal co-elution of host proteins or buffer components. Although we did not perform direct inhibitor quantification in this study, qPCR amplification efficiency and electrophoresis results indicate that these trace co-elutes did not inhibit downstream detection. Specifically, M3 templates consistently produced the earliest Ct values and the highest plateau fluorescence in qPCR (Fig 1C–G), and M3 yielded 2.8-fold more amplifiable template than M1 (Fig 1I), confirming that the slight spectrophotometric impurity does not adversely affect PCR. In contrast, while M1/M2 achieved more “ideal” spectrophotometric ratios through repeated centrifugation and buffer exchanges, this came at the cost of template loss—an acceptable trade-off for high-concentration samples but a critical drawback for low-density infections where every intact template molecule counts. In the present study, the method with the highest functional recovery (M3) detected 77.5% of low-density clinical samples, compared to only 60.0% for M1, despite M3 having the lower purity ratio. This finding indicates that, for low-density *P.* *falciparum* screening, functional performance metrics—such as yield, integrity, and amplification efficiency—warrant careful consideration alongside spectrophotometric purity. Future studies incorporating dedicated protein assays or PCR inhibition testing would provide a more complete characterization of extract purity.

Another interesting finding from the dilution experiments was that for Sample 3, both M1 and M3 produced clear amplification at the 10^4^ dilution (24.4 parasites/μL), whereas M1 failed at similar dilution levels in other samples. This sample-specific discrepancy likely reflects the inherent heterogeneity of clinical specimens. Variables such as white blood cell count, hemoglobin content, or residual drug metabolites may differentially affect the adsorption and elution efficiency of silica columns, leading to inconsistent performance. In contrast, the consistent superiority of M3 across all samples underscores its robustness against such matrix variability—a key attribute for reliable low-density detection.

When considering both performance and cost, the cost-effectiveness of each method is a practical concern. Based on market data for comparable commercial kits, the per-extraction reagent cost is approximately $3.8–5.2 for silica columns (M1), $4.2–5.5 for manual magnetic bead methods, and significantly higher ($9.5–11.0) for automated magnetic bead systems. Although M3 has slightly higher reagent costs, its enhanced sensitivity makes it a worthwhile investment for high-stakes surveillance, where a missed low-density infection could lead to undetected resistance clusters and wider transmission. We acknowledge that only three extraction methods were compared. Other commonly used methods, such as Qiagen QIAamp kits (approximately $8.77 per sample) and Chelex-100 extraction, were not included. Qiagen kits share the same silica membrane column principle as M1 but at a higher cost, so M1 was selected as the representative of this category. Chelex-100 is based on metal chelation and boiling lysis, a principle fundamentally different from the solid-phase extraction techniques evaluated here. We recognize that not including these methods limits direct comparison with some published studies. Future studies should include a broader range of methods to further guide method selection for low-density malaria surveillance.

While this study systematically evaluated three DNA extraction methods for *P. falciparum* and clarified the advantages of magnetic beads for low-density detection, certain limitations remain. First, the focus was primarily on *P. falciparum*. In malaria elimination settings, particularly in the Asia-Pacific region, *Plasmodium vivax* also poses a significant challenge. *P. vivax* has a hypnozoite stage, and the characteristics of its low-density infections—as well as the requirements for extraction methods—may differ. Whether our conclusions can be directly extrapolated to *P. vivax* requires further validation. Second, the sample sizes in this study were relatively modest: 10 samples for the initial method comparison, 4 for the dilution series, and a clinical validation cohort of 40 post-treatment patients from a single center. Samples with low residual parasitemia after ACT treatment are scarce locally, and with China now in the malaria post-elimination phase, access to *P. falciparum*-positive blood has become inherently limited. Future studies should incorporate larger, multi-center cohorts with diverse epidemiological settings, including *P. vivax* and mixed-species infections, to further strengthen generalizability. Third, the parasite densities used for the serial dilution sensitivity experiments were determined by a single WHO-certified Level 1 malaria microscopist. To reduce random error, each slide was counted three times and the mean was used. Nevertheless, the absence of an independent second reader remains a limitation, as a double-read protocol with discrepancy resolution would have provided stronger quality control. Future studies should adopt a double-read plus tie-breaker protocol to further strengthen microscopy-based quantitation.

Based on our findings, future work could proceed along the following lines: First, evaluate the extraction performance of magnetic bead methods on sample types more suitable for field collection and transport, such as RDT strips or dried blood spots (DBS), to support molecular surveillance in resource-limited settings. Second, systematically apply this approach in prospective studies on ACT efficacy monitoring and resistance early warning. By accurately detecting post-treatment submicroscopic parasitemia and correlating it with parasite clearance kinetics and molecular resistance markers, such research could provide critical evidence for establishing an early warning system for drug resistance based on high-sensitivity nucleic acid extraction, thereby enhancing proactive surveillance of treatment failure and resistance risk.

## Supporting information

S1 Table Comparison of DNA concentrations extracted from 10 *P. falciparum*-positive whole blood samples using M1, M2, and M3. (DOCX)

S2 Table Comparison of A_260_/A_280_ ratios for DNA extracted by the three methods. (DOCX)

S3 Table Relative template recovery efficiency (2^–ΔCt^) calculated from qPCR Ct values, with M2 set as the baseline (value of 1). (DOCX)

S4 Table Quantitative comparison of relative template recovery efficiency (2-ΔCt), calculated from qPCR Ct values with the 10-minute lysis group set as the baseline. (DOCX)

S5 Fig Original, uncropped gel images corresponding to Fig 4A for samples S1–S6. (TIF)
