# Supplementary material for: Magnetic bead-based enhancement of qPCR detection rates in low-density Plasmodium falciparum samples: A comparative analysis of nucleic acid extraction methods
Source: PLoS One. 2026 Aug 6;21(8):e0355288. doi: 10.1371/journal.pone.0355288 (PMC13446695; doi:10.1371/journal.pone.0355288)
Supplement: Supplementary file 1 [file pone.0355288.s001.docx]

**S1**

**Figure1, A**

Comparison of DNA concentrations extracted from 10 *P. falciparum*-positive whole blood samples using M1, M2, and M3

| Sample | M1 | M2 | M3 |
| --- | --- | --- | --- |
| S1 | 10.1 | 7.7 | 40.3 |
| S2 | 12.8 | 9.9 | 56.5 |
| S3 | 11.9 | 9.5 | 32.6 |
| S4 | 8.8 | 6.4 | 29.8 |
| S5 | 9.6 | 8.2 | 44.3 |
| S6 | 7.1 | 5.9 | 33.6 |
| S7 | 7.7 | 6.7 | 31.7 |
| S8 | 7.9 | 5.1 | 38.5 |
| S9 | 12.9 | 5.4 | 38.5 |
| S10 | 16.1 | 10.0 | 44.4 |
