# Supplementary material for: Magnetic bead-based enhancement of qPCR detection rates in low-density Plasmodium falciparum samples: A comparative analysis of nucleic acid extraction methods
Source: PLoS One. 2026 Aug 6;21(8):e0355288. doi: 10.1371/journal.pone.0355288 (PMC13446695; doi:10.1371/journal.pone.0355288)
Supplement: Supplementary file 2 [file pone.0355288.s002.docx]

**S2**

**Figure1, B**

Comparison of A₂₆₀/A₂₈₀ ratios for DNA extracted by the three methods.

| Sample | M1 | M2 | M3 |
| --- | --- | --- | --- |
| S1 | 1.74 | 1.75 | 1.60 |
| S2 | 1.77 | 1.64 | 1.76 |
| S3 | 1.75 | 1.77 | 1.64 |
| S4 | 1.75 | 1.69 | 1.66 |
| S5 | 1.77 | 1.79 | 1.69 |
| S6 | 1.75 | 1.68 | 1.66 |
| S7 | 1.71 | 1.32 | 1.64 |
| S8 | 1.79 | 1.67 | 1.70 |
| S9 | 1.78 | 1.77 | 1.68 |
| S10 | 1.75 | 2.55 | 1.71 |
