# Supplementary material for: Magnetic bead-based enhancement of qPCR detection rates in low-density Plasmodium falciparum samples: A comparative analysis of nucleic acid extraction methods
Source: PLoS One. 2026 Aug 6;21(8):e0355288. doi: 10.1371/journal.pone.0355288 (PMC13446695; doi:10.1371/journal.pone.0355288)
Supplement: Supplementary file 3 [file pone.0355288.s003.docx]

**S3**

**Figure1, I**

Relative template recovery efficiency (2^–ΔCt^) calculated from qPCR Ct values, with M2 set as the baseline (value of 1)

| Sample | M1 | M2 | M3 |
| --- | --- | --- | --- |
| S1 | 1.46 | 1 | 4.20 |
| S2 | 1.72 | 1 | 5.43 |
| S3 | 1.50 | 1 | 2.38 |
| S4 | 1.68 | 1 | 4.00 |
| S5 | 1.27 | 1 | 4.11 |
| S6 | 1.16 | 1 | 3.86 |
| S7 | 1.23 | 1 | 3.34 |
| S8 | 1.31 | 1 | 5.43 |
| S9 | 1.81 | 1 | 4.53 |
| S10 | 1.30 | 1 | 2.73 |
