# Supplementary material for: Magnetic bead-based enhancement of qPCR detection rates in low-density Plasmodium falciparum samples: A comparative analysis of nucleic acid extraction methods
Source: PLoS One. 2026 Aug 6;21(8):e0355288. doi: 10.1371/journal.pone.0355288 (PMC13446695; doi:10.1371/journal.pone.0355288)
Supplement: Supplementary file 4 [file pone.0355288.s004.docx]

**S4**

**Figure2, C**

Quantitative comparison of relative template recovery efficiency (2^-ΔCt^), calculated from qPCR Ct values with the 10-minute lysis group set as the baseline.

| Sample | 10min | 30min | 60min | 120min |
| --- | --- | --- | --- | --- |
| 1 | 1 | 1.19 | 1.55 | 0.92 |
| 2 | 1 | 1.21 | 1.60 | 0.93 |
| 3 | 1 | 1.22 | 1.48 | 0.93 |
| 4 | 1 | 0.97 | 0.99 | 1.40 |
| 5 | 1 | 0.99 | 1.05 | 1.08 |
| 6 | 1 | 0.91 | 0.97 | 1.29 |
